# Miscellaneous

**Published:** 1876-05

**Authors:** 


### Miscellaneous.

### AVOIDANCE OF PHOSPHORUS POISONING.

At the Brussels Congress there was presented the follow-report of M. Crocq, "On the Sanitary Measures in Workshops where Phosphorus is Manipulated." The following conclusions were adopted: The section of public medicine expresses the wish, 1. That the use of red amorphous phosphorus be substituted for that of ordinary phosphorus in all match factories. 2. Until the universal adoption of this radical measure, it recommends, in the actual conditions of manufacturing, the following measures, which are designed to prevent general toxic accidents, and more especially maxillary necrosis; installations of the manufacturing in sufficiently spacious rooms; powerful ventilation promoted by the aid of tubes, beginning at the ground and terminating in a drawing chimney; constant attention to cleanliness; together with these physical means, use as a chemical antidote, the spirits of turpentine. 3. Local accidents may be averted by astringent gargarisms, and above all, by the obligation imposed upon manufacturers to admit none into their workshops, who, by oral examination, present dental lesions, such as penetrating decay, or any other affection of a nature to favor the deleterious action of phosphoric vapors. 4. Children not to be employed in workshops where phosphorus is used. 5. When the authorities allow the establishment of manufactories where that substance is used, they must impose these conditions and see that they are observed, for the interests of workmen as well as of manufacturers, who are criminally responsible for accidents resulting from their carelessness or neglect.

---

### THE SKIN AND BODILY TEMPERATURE.

The following conclusions are given by Dr. Winternitz on the importance of the cutaneous function with regard to the heat of the body. His paper is given in Stricher's *Jahrbucher*.

1. The increase and diminution of loss of heat from the skin can be stated approximately in figures.

2. Experiments show that the giving off of heat may vary more than 60° downward, and more than 92° upward.

3. Such a variation in loss of heat can compensate variations in the production of heat three times the normal amount.

4. The discoverable variations in the loss of heat suffice to explain the consistency of temperature, so far as it exists under the usual cooling and warming influences.

5. The diminution in the loss of heat, consequently its retention, after previous loss, even if the production remain constant, is sufficient to replace in a short time the loss of temperature.

6. A diminution in the loss of heat alone can in many cases explain a febrile rise of temperature.

7. The possible increased loss of heat of more than 92° explains the frequently observed rapid disappearance of fever.

8. There is, consequently, no doubt that one of the most important factors in the regulation of tempetrature is to be found in the function of the skin.

### KENTUCKY STATE DENTAL SOCIETY.

The annual meeting of the Kentucky State Dental Society will be held in Louisville, on the first Tuesday in June, prox.

We print below the programme of subjects for discussion. They are all important, and will doubtless elicit interesting and instructive discussions. It is very desirable that the profession of the State be well represented, and the profession outside of the State have a cordial invitation.

### PROGRAMME.

1. Inflammation and absorption of the gums and alveolar process.
2. Relation of Dentistry to Medicine.
3. Necrosis of the maxillary bones. Its cure and effect, and treatment.
4. Deciduous teeth, their relation to the permanent teeth, and their treatment.
5. Transplanting and replanting of teeth.
6. Exposed nerves; devitalized teeth; alveolar abscess and treatment.
7. Facial neuralgia and treatment.
8. Relative value of rubber and celluloid.
9. Relative merits of gold for filling teeth, with demonstrations. Cylinder filling, by W. G. Redman.

CHAS. E. DUNN, Pres.

A. O. RAWLS, Sec.

### CONNECTICUT VALLEY DENTAL SOCIETY.

The semi-annual meeting of the Connecticut Valley Dental Society, for 1876, will be held in Stedman Hall, No. 3

Pratt Street, Hartford, Conn., on Tuesday and Wednesday, June 13th and 14th, commencing at 2 p. m. on Tuesday.

QUESTIONS FOR DISCUSSION.

1. Mechanical Dentistry.
2. Treatment of Exposed Pulps in Deciduous Teeth.
3. Anæsthesia.
4. Fungi—What are its Effects upon the Teeth.
5. Causes of Dental Decay.
6. The Rise and Progress of Dentistry in U. S. A.—Essay by Dr. C. S. Hurlbut.
7. Use and Abuse of the Dental Engine.
8. Miscellaneous Subjects.

The evening session will be devoted to a Lecture by Prof. J. E. Garretson, M. D., of Philadelphia. Subject: "The Clinical Signification and Treatment of Tumors of the Mouth, Jaws and Face."

Gentlemen having volunteer Essays are requested to notify the Chairman of the Executive Committee, Dr. L. C. Taylor.

A general invitation is cordially extended to all members of the profession to be present at this meeting.

OFFICERS OF THE SOCIETY.

President, H. F. Bishop, Worcester, Mass.
1st Vice-President, H. W. Clapp, Westfield, Mass.
2d Vice-President, E. M. Goodrich, Westfield, Mass.
Secretary, C. T. Stockwell, Springfield, Mass.
Treasurer, N. Morgan, Springfield, Mass.

EXECUTIVE COMMITTEE.

L. C. Taylor, Hartford, Conn.
J. N. Davenport, Northampton, Mass.
L. Noble, Springfield, Mass.

C. T. STOCKWELL, Secretary.

SPRINGFIELD, MASS., April 21, 1876.

### IOWA STATE DENTAL SOCIETY.

Thirteenth annual session of the Iowa State Dental Society will be held at Burlington, May 16th.

### OFFICERS.

C. P. Artman, President.
P. T. Smith, Vice-President.
R. L. Cochran, Rec. Sec'y.
I. P. Wilson, Cor. Sec'y

### EXAMINING COMMITTEE.

J. T. Abbott, J. S. Shattuck, L. J. Waters.

### EXECUTIVE COMMITTEE.

L. C. Ingersoll, J. B. Morgan, Joseph Kulp.

### PUBLICATION COMMITTEE.

W. P. Dickinson, W. Hawks, Dr. Fuller.

### PROGRAMME.

The Society will convene on Tuesday morning, May 16th, at 9 o'clock, and close its sessions on Thursday evening.

Members are requested to be promptly present.

The Examining Committee will meet on Monday evening previous, for examination of applicants for membership, at the office of Dr. I. P. Wilson.

Tuesday forenoon will be devoted to business.

Clinics will occupy the forenoon of Wednesday.

### CLINICAL OPERATORS.

G. H. Cushing, of Chicago; Wm. O. Kulp, of Davenport; L. C. Ingersoll, of Keokuk.

Wednesday evening, a popular lecture on Dentistry, will be delivered by Dr. M. S. Dean, of Chicago.

Members and others, who have any interesting cases in model or drawings, are requested to bring them; also valuable instruments of new patterns.

Hotel accommodations reduced to $2.00 per day.

Members who go by steamer, paying full fare, will be returned at one-third rate by obtaining cards of membership from the Recording Secretary.

ESSAYISTS AND SUBJECTS.

Fractures of the Inferior Maxilla. A. J. Waid, Newton.

Professional Attainment. A. V. Eaton, Anamosa.

Essential Elements of Success in Dental Practice. K. B. Davis, Petersburg, Ill.

Facts. W. H. Barker, Pella.

Dental Pathology. Gust. North, Springville.

Care of Children's teeth. S. L. Edwards, Des Moines.

Physiology and Hygiene. J. F. Sanborn, Tabor.

The Degeneracy of Human Teeth. J. Hardman, Muscatine.

Is the Degree of D. D. S., only a "Badge of Partial Culture?" J. B. Morgan, Davenport.

Our Mission. I. P. Wilson, Burlington.

Responsibility of Parentage, Physiologically Considered. J. Thompson, Sigourney.

Voluntary Essays; Reports of Cases, etc.

It is important that those who expect to attend the meeting of the Society, should come regarding it as an established SCHOOL of the profession—an Educational Institution where all are teachers, and all are learners. Coming together in this spirit, and into this mutal relation, it is incumbent upon every member to look over the list of subjects to be presented in the essays, and select one or more upon which he will bestow thought and study, and thus be prepared when the subject is thrown open for discussion to express his thoughts and opinions in a concise and lucid manner. By this means we shall avoid random talk, and make our remarks worthy of consideration.

L. C. INGERSOLL,<br>

J. B. MORGAN, } Ex. Com.<br>

JOSEPH KULP.

